# Lipedema or Advanced Cellulite? A Diagnostic Boundary in Need of Definition

**DOI:** 10.1111/jocd.71056

**Published:** 2026-07-07

**Authors:** Dora Intagliata, Maria Luisa Garo

**Affiliations:** ^1^ Poliambulatorio ID Future Siracusa Italy; ^2^ Biostatistics and Research Methodology Unit Mathsly Research Vibo Valentia Italy

## 1 Introduction

Lipedema and advanced cellulite are pathophysiologically distinct entities that share an anatomical territory. Lipedema is a chronic disorder of subcutaneous adipose tissue with emerging genetic, hormonal, and inflammatory underpinnings [1, 2, 3]. Cellulite is a multifactorial subcutaneous condition involving adipose hypertrophy, fibrous reorganization of the dermal–hypodermal interface, microcirculatory impairment, and a variable edematous component [4], affecting most post‐pubertal women regardless of body weight [5, 6]. In a subset of patients, typically those with mild‐to‐moderate lipedema, they can produce remarkably similar clinical features, including palpable nodularity, irregular surface texture, increased subcutaneous thickness, and tenderness on palpation. Notably, the standard differential diagnoses canonically considered against lipedema are obesity, lymphedema, lipohypertrophy, chronic venous insufficiency, and rarer fatty‐deposition syndromes [3, 7, 8, 9, 10] not considering advanced cellulite at all.

Both entities are diagnosed clinically. The recent Delphi consensus of the Lipedema World Alliance states explicitly that no imaging, serological, or genetic test, and no clinical measurement instrument, is currently approved to verify the diagnosis of lipedema [11]; instrument‐based modalities, including high‐resolution ultrasound, are assigned to the exclusion of differential diagnoses [7, 9, 10]. For the typical lipedema presentation, clinical criteria are discriminating and, in experienced hands, dependable [12, 13, 14]. The same holds for advanced cellulite, characterized and graded through the Nürnberger–Müller classification, the validated Cellulite Severity Scale [15, 16], and, more recently, ultrasound‐based staging systems [17].

However, in mild‐to‐moderate lipedema, the phenotype converges with that of advanced fibrotic cellulite, and the two conditions cannot be reliably distinguished on the available clinical and imaging criteria. Under these conditions, the omission of advanced cellulite from standard lipedema differential lists ceases to be a taxonomic oversight and becomes a clinical problem with direct therapeutic implications.

## 2 Where the Phenotypes Converge

The diagnostic difficulty arises in the overlap zone between mild‐to‐moderate lipedema and moderate‐to‐severe fibrotic cellulite. The features used to define lipedema are also observed in advanced fibrotic cellulite. Even the most reliable discriminators of lipedema—bilateral symmetry, easy bruising, and foot sparing—may not clearly separate the two conditions. Notably, the most recent German S2k guideline has explicitly removed nodularity from the diagnostic criteria of lipedema on grounds of insufficient validity [9], since palpable nodularity is also shared with fibrotic cellulite.

Among the other features, pain occupies a special place. The same guideline elevates pain from an auxiliary sign to a defining criterion: lipedema is to be described as a painful disproportionate symmetric distribution of adipose tissue, and a disproportionate adipose increase without corresponding symptoms is to be classified not as lipedema but as lipohypertrophy [9]. Pressure pain, tenderness, spontaneous pain, and a sensation of heaviness are the cardinal symptoms [9]. Yet this same constellation is also characteristic of advanced fibrotic cellulite, in which a low‐grade inflammatory milieu in gluteofemoral adipose tissue has been proposed as the substrate of tenderness [4].

The convergence extends beyond the clinical and into the pathophysiological literature which propose that cellulite and lipedema may reflect consecutive stages of a single process in gluteofemoral adipose tissue, driven by selective endotoxemia and progressive adipose remodeling [4].

## 3 Why Imaging Does Not Resolve the Ambiguity

If clinical criteria reach their limit in the overlap zone, one might expect imaging or histology to resolve the ambiguity. The available evidence suggests otherwise. The morphological features attributed to lipedema and cellulite are neither specific to either condition nor reproducibly detected. A large sonomorphological study of lipedema found that the septa‐rich subcutis classically described in the lipedema literature could not be confirmed on blinded re‐reading: septal patterns and cloudy echogenicity were non‐specific and did not correlate with the clinical diagnosis [18]. Aksoy et al. similarly noted that duplex ultrasound examination is not specific in lipedema [7]. Naouri et al., using 20 MHz high‐resolution ultrasonography, showed that dermal thickness and echogenicity in lipedema are indistinguishable from healthy controls; the technique discriminated lymphedema from lipedema, but did not identify a positive lipedema signature [19].

Histology reinforces this picture rather than resolving it. Felmerer et al. found no significant difference in dermal collagen deposition between lipedema patients and BMI‐matched controls (Sirius red staining), although adipose tissue itself showed increased fibrosis and adipocyte hypertrophy [20]. Barros et al., in a small case series of 12 women with clinically diagnosed Stage II–III lipedema reported septal fibrosis in only 10% of biopsies [21].

Collectively, the available imaging and histological tools do not provide a positive signature for lipedema; they provide, at best, exclusion of alternative diagnoses such as lymphedema. A recent scoping review of assessment modalities for lower‐extremity edema, lymphedema, and lipedema [22] reaffirms that there is no single definitive diagnostic test for lipedema, while restricting its comparative scope to lymphedema and generalized edema; advanced cellulite is not considered as a differential at any point in the review. The comparator against which lipedema imaging is routinely tested is lymphedema, never cellulite.

## 4 The Clinical Stakes of Misclassification

Why does the diagnostic boundary matter? Because the two conditions are managed along sharply divergent therapeutic paths from the first intervention. A patient classified as having advanced fibrotic cellulite is, as first‐line management, a candidate for procedures targeting the dermal–subcutaneous fibrous architecture. A patient classified as having lipedema is steered toward an altogether different paradigm. Consensus and guideline documents for lipedema [7, 8, 9, 10, 11, 14] converge on conservative management as first‐line: compression at stage‐adjusted pressures, complex decongestive therapy, and weight and lifestyle optimization. When conservative measures fail to control pain or functional impairment, liposuction, performed with tissue‐ and lymph vessel‐conserving techniques, is recommended as the surgical method of choice for sustainable reduction of the affected subcutaneous adipose tissue [9]; it does not cure the condition but may permanently alleviate pain and improve quality of life. Pharmacological caution is also recommended: certain medication classes such as glucocorticoids, thiazolidinediones, thiazide and loop diuretics are generally avoided in lipedema given the risk of exacerbating edema or promoting local fibrosclerosis [3].

The genetic, hormonal, and inflammatory underpinnings now emerging for lipedema [1, 2] reinforce that lipedema is a systemic disorder of adipose tissue, not a localized architectural defect amenable to septum‐directed correction. A patient steered down the wrong therapeutic path therefore does not simply receive a suboptimal intervention; she receives one premised on a fundamentally different model of her tissue. The mismatch runs in both directions.

A patient with unrecognized lipedema routed down the cellulite‐directed path is subjected to subcision or septal‐lysis devices in a tissue compartment whose lymphatic system, while not obstructed as in lymphedema, is functionally vulnerable and at risk of iatrogenic injury: a vulnerability that the cellulite‐directed procedural framework does not register. Conversely, a patient with advanced fibrotic cellulite labeled as lipedema is committed to a long‐term conservative framework that addresses neither the dermal–hypodermal architectural substrate of her condition nor its localized fibrotic remodeling. The lipedema‐conservative framework, sustained over years, addresses a different pathophysiology; and even if her persistent symptoms eventually prompt liposuction under the lipedema‐refractory‐pain criterion [9], this too would be the wrong instrument for a condition whose substrate is fibrotic remodeling of the dermal–hypodermal interface rather than diffuse adipose tissue expansion.

Which therapeutic path a borderline patient follows in current practice may therefore depend less on the biology of her tissue than on whether she first consults an aesthetic physician or a phlebologist. A diagnostic boundary that routes patients to divergent treatments on the basis of referral pathway rather than pathophysiology is one that demands clarification.

## 5 Toward a Diagnostic Framework

The remedy is not to retreat from clinical diagnosis, which serves the typical patient well, but to supplement it where it is weakest. Three steps would begin to close the gap. First, advanced cellulite should be explicitly added to the differential diagnoses considered for women presenting with disproportionate, painful, and tender lower‐limb subcutaneous changes. Its current absence from standard differential lists is itself a source of diagnostic bias [7, 8, 9, 10]. The proposal is not without precedent: A 2022 study has argued for the routine consideration of cellulite alongside lipedema, obesity, and idiopathic cyclic edema as one of four causes of increased lower‐limb volume, noting that some 80% of women with lipedema also present features of cellulite [23], a position so far isolated within the lipedema literature.

Second, imaging research should be reframed to directly compare lipedema with advanced cellulite rather than only with lymphedema or healthy controls: a comparison the diagnostic accuracy literature has so far almost entirely overlooked. Candidate discriminators would include the morphology and distribution of fibrous septa, dermal echogenicity at standardized sites, subcutaneous compressibility, and microvascular flow patterns [21], assessed against the established clinical diagnosis as the reference standard. Cross‐sectional studies in clinically unambiguous patients of each condition would be a necessary first step, followed by validation in borderline cases.

Third, until such a framework exists, the working clinical posture in borderline cases should be one of explicit uncertainty rather than default categorization. Patients should be informed that the picture is ambiguous, that the available evidence does not yet allow a confident assignment, and that therapeutic decisions should be made conservatively until clearer criteria become available.

## 6 Conclusion

Lipedema and advanced cellulite are pathophysiologically distinct conditions that occupy the same anatomical compartment and, in a subset of patients, the same phenotypic territory. Where their presentations converge, neither clinical criteria nor available imaging is calibrated to distinguish them, and the therapeutic paths to which the two labels lead diverge sharply. Closing this gap will require explicit acknowledgement of advanced cellulite in the differential diagnosis of lipedema, comparative imaging studies designed to test it, and a clinical posture of measured uncertainty in borderline cases.

## Conflicts of Interest

The authors declare no conflicts of interest.

## Data Availability

Data sharing not applicable to this article as no datasets were generated or analyzed during the current study.
